# 1341. Clinico-demographic Features and Mortality of COVID-19 Patients in Southwest Michigan

**DOI:** 10.1093/ofid/ofad500.1178

**Published:** 2023-11-27

**Authors:** Alana Pinheiro Alves, Alec Johnson, Benjamin I Bizer, Matthew Belardo, Kathryn Garber, Mujahed Abbas

**Affiliations:** Western Michigan University, Kalamazoo, Michigan; Western Michigan University, Kalamazoo, Michigan; Western Michigan University, Kalamazoo, Michigan; Western Michigan University, Kalamazoo, Michigan; Western Michigan University, Kalamazoo, Michigan; Western Michigan University, Kalamazoo, Michigan

## Abstract

**Background:**

Since the beginning of the COVID-19 pandemic to May 2023, Michigan has had over 3 million confirmed or probable disease cases, with nearly 43 thousand deaths. Southwestern Michigan was significantly impacted and contributed to a high burden of the disease in the state. Our study objectives were to describe mortality rates and some of the demographic and clinical characteristics of COVID-19 in this area.

**Methods:**

This is a retrospective cohort analysis in one community hospital in Kalamazoo County, Southwestern Michigan, United States. Institutional Review Board approval was obtained. Data was extracted from electronic medical records (EMR). Patients admitted to the hospital from March 2020 to December 2022 with a positive polymerase chain reaction (PCR) test for SARS-CoV-2 were included. Pregnant patients and those less than 18 years of age were excluded.

**Results:**

A total of 426 patients were included in this study, mainly males. The median age of hospitalization was 65 years. The most prevalent co-morbidities were type 2 diabetes mellitus, hypertension, and coronary artery disease. The median length of hospital stay was ten days. 139 (32,6%) individuals died, the majority of whom were men aged between 50 and 80. At the same time, we noted higher death rates among American Indian or Alaskan Native individuals and Black or African American patients. Review Figures 1 and 2 for further details.

**Figure:**
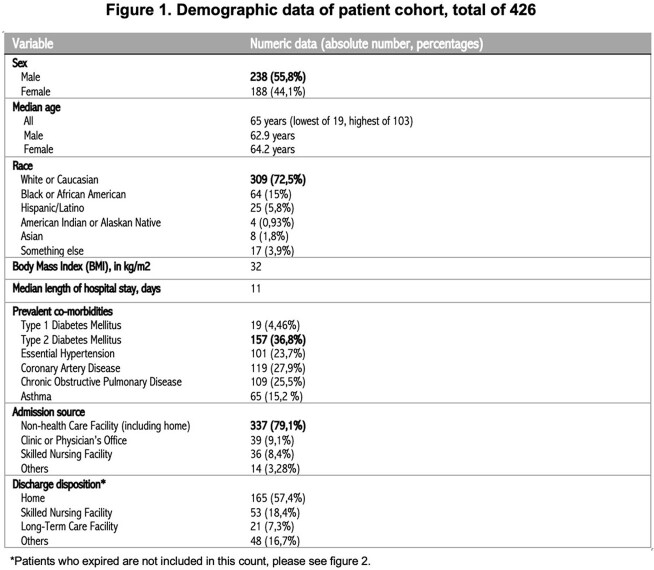

**Figure:**
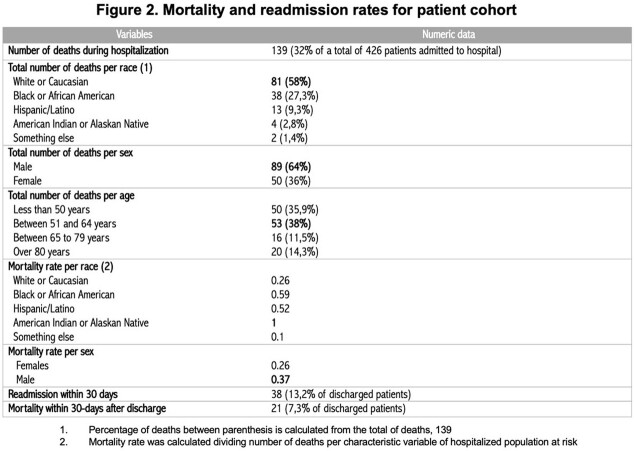

**Conclusion:**

A high absolute number of deaths among Whites or Caucasians was noted, likely related to local demographics where most of the population self-identifies as so. They were the most prevalent group of hospitalized individuals in our cohort. All four American Indian and Alaskan Native individuals included in the results died. Like national and state data, Black or African Americans and Hispanic/Latinos had a higher death rate than Whites. Variables such as co-morbidities, length of stay, and need for mechanical ventilation within these groups were not considered when calculating such rates. A 32,6% percent of deaths in our cohort is more elevated than local and worldwide statistics. However, this is a small study with several confounding factors that lacked calculation of statistical significance.

**Disclosures:**

**All Authors**: No reported disclosures

